# Novel insights into vascular dysfunction in cuprizone-induced demyelination through functional ultrasound imaging

**DOI:** 10.1162/imag_a_00534

**Published:** 2025-04-10

**Authors:** Benoit Beliard, Lauriane Delay, Youenn Travert-Jouanneau, Nathalie Ialy-Radio, Célia Isaad, Annabelle Réaux-Le Goazigo, Thomas Deffieux, Daniel P. Bradley, Mickael Tanter, Sophie Pezet

**Affiliations:** Physics for Medicine Paris, Inserm, ESPCI Paris, CNRS, PSL Research University, Paris, France; Sorbonne Université, INSERM, CNRS, Institut de la vision, Paris, France; Biogen, Cambridge, MA, United States

**Keywords:** neuroimaging, neurovascular coupling, blood flow, myelin basic protein, primary sensory cortex, rise time

## Abstract

Multiple Sclerosis (MS) is an autoimmune disease of the central nervous system (CNS), affecting 2.8 million people worldwide, that presents multiple features, one of which is demyelination. Although treatments exist to manage the condition, no cure has been found to stop the progression of neurodegeneration. To develop new treatments and investigate the multiple systems impacted by MS, new imaging technologies are needed at the preclinical stage. Functional ultrasound imaging (fUS) has recently emerged as a robust method to measure brain cerebral blood volume (CBV) dynamics as an indirect indicator of neural activity. This study aimed to quantify the amplitude of alteration of evoked hemodynamic response in the somatosensory cortex, and its potential link with demyelination in a mouse model of CNS demyelination induced by cuprizone. We demonstrate that extended demyelination leads to an increased hemodynamic response in the primary sensory cortex, both spatially and temporally, aligning with fMRI findings in MS patients. Second, using descriptors of the evoked cortical hemodynamic response, we demonstrate that certain parameters (the number of active pixels and the rise time) correlate with the level of Myelin Basic Protein in the primary sensory cortex and the thalamus, when taken together. Interestingly, the increased CBV is not associated with demyelination but instead reflects the well-documented vascular alteration described in MS. Moreover, these changes were absent in the thalamus, and in focalized demyelinated lesions induced by lysolecithin injection, suggesting the involvement of specific cortical mechanisms driven by oligodendrocyte depletion. In conclusion, our study introduces a novel, non-invasive functional approach for investigating vascular dysfunction in the context of MS, addressing an important yet understudied aspect in both pre-clinical and clinical research.

## 1 Introduction

Multiple sclerosis (MS) is an autoimmune neurodegenerative disorder of the central nervous system. It is the most common cause of neurological disability in young adults, affecting approximately 2.8 million worldwide (Ramagopalan et al., 2010). Chronic inflammation, demyelination, gliosis, and neuronal loss are hallmarks of the pathology, producing plaque formation and tissue destruction, in both white and grey matter (Garg & Smith, 2015;Lassmann, 2013). Magnetic Resonance Imaging (MRI) is largely used for MS diagnosis and follow up, but to date, it is not able to distinguish among MS disease courses. Preclinical studies are crucial for understanding these mechanisms and developing new therapeutics. Models of demyelination, such as cuprizone, have been pivotal in validating novel imaging techniques, including magnetization transfer ratio (Tagge et al., 2016), diffusion tensor imaging (Hübner et al., 2017;Sun et al., 2006), and macromolecular proton fraction mapping (M. Khodanovich et al., 2019;M. Y. Khodanovich et al., 2017). Several studies have even utilized multimodal neuroimaging in this model to evaluate the sensitivity and specificity of these techniques, with electron microscopy serving as the reference for anatomical changes (Ding et al., 2021;Friesen et al., 2024;Wood et al., 2016). Among emerging imaging approaches, hyperpolarized 13C magnetic resonance spectroscopic imaging shows promise for assessing inflammatory lesions (Guglielmetti et al., 2017), while TSPO-[18F]DPA-714 PET enables the imaging of proinflammatory cells, such as astrocytes and microglia, across multiple brain regions (Zinnhardt et al., 2019). Furthermore, NIRS-MRI facilitates the quantification of the reduced cerebral metabolic rate (Hashem et al., 2022).

Despite these advances, few studies have examined the functional consequences of demyelination within neural networks (Cerina et al., 2017;Narayanan et al., 2018). While fMRI studies in MS patients have demonstrated abnormal evoked hemodynamic responses, no preclinical study has yet explored this critical aspect.

Ultrafast ultrasound imaging is a relatively new imaging technology that detects changes of cerebral blood volume with a great sensitivity (Deffieux et al., 2021;Mace et al., 2013), allowing functional studies in both anesthetized (Claron et al., 2021;Mace et al., 2011) and awake animals (Bimbard et al., 2018;Matei et al., 2022), the study of functional connectivity (Osmanski et al., 2014;Rahal et al., 2020), of microvascular flux at a microscopic scale with a large field of view (Errico et al., 2015;Réaux-Le-Goazigo et al., 2022), and finally functional studies at a microscopic scale (Renaudin et al., 2022).

Given the scarcity of preclinical studies investigating functional hyperemia in animal models of CNS demyelination, this study aimed to investigate alterations in cortical hemodynamic response to sensory stimulation in a widely used animal model of CNS demyelination. Cuprizone (bis-cyclohexanone-oxalyldihydrazone, CPZ), a copper chelator, induces chronic (non-immune) demyelination of the brain by targeting oligodendrocytes (Matsushima & Morell, 2001;Praet et al., 2014;Vega-Riquer et al., 2019). Its withdrawal facilitates the study of natural remyelination and its enhancement via pharmacological interventions. Given the extensive sensory innervation of the whisker pad in rodents (Petersen, 2019), we chose the barrel cortex as a model system. Our study demonstrates long-lasting alterations of the hemodynamic response in cuprizone-induced demyelinated animals, and three descriptors of this evoked response correlate with the level of Myelin Basic Protein in several key brain areas involved in sensory processing. Our results also suggest disrupted vascular responsiveness, paralleling clinical observations from the early phase of the disease.

## 2 Materials and Methods

### 2.1 Compliance with Ethical Guidelines

All experiments performed in this study complied with the French and European Community Council Directive of September 22 (2010/63/UE). They were also approved by the local Institutional Animal Care and Ethics Committees (#59, ‘Paris Centre et Sud’ project #2019-41). In adherence to these guidelines, the number of animals in our study was kept to the minimum necessary. Using previously published data and preliminary data on the effect of cuprizone treatment, we performed a G-Power analysis (https://www.psychologie.hhu.de/arbeitsgruppen/allgemeine-psychologie-und-arbeitspsychologie/gpower) and determined that N = 8 mice per group were sufficient to detect statistical differences in our imaging experiments.

### 2.2 Sex as a biological variable

Our study examined male mice only because male animals exhibited less variability in phenotype. It is unknown whether the findings are relevant for female mice. Therefore, this is one of the limitations of this study.

### 2.3 Experimental design

**Fig. 1.**
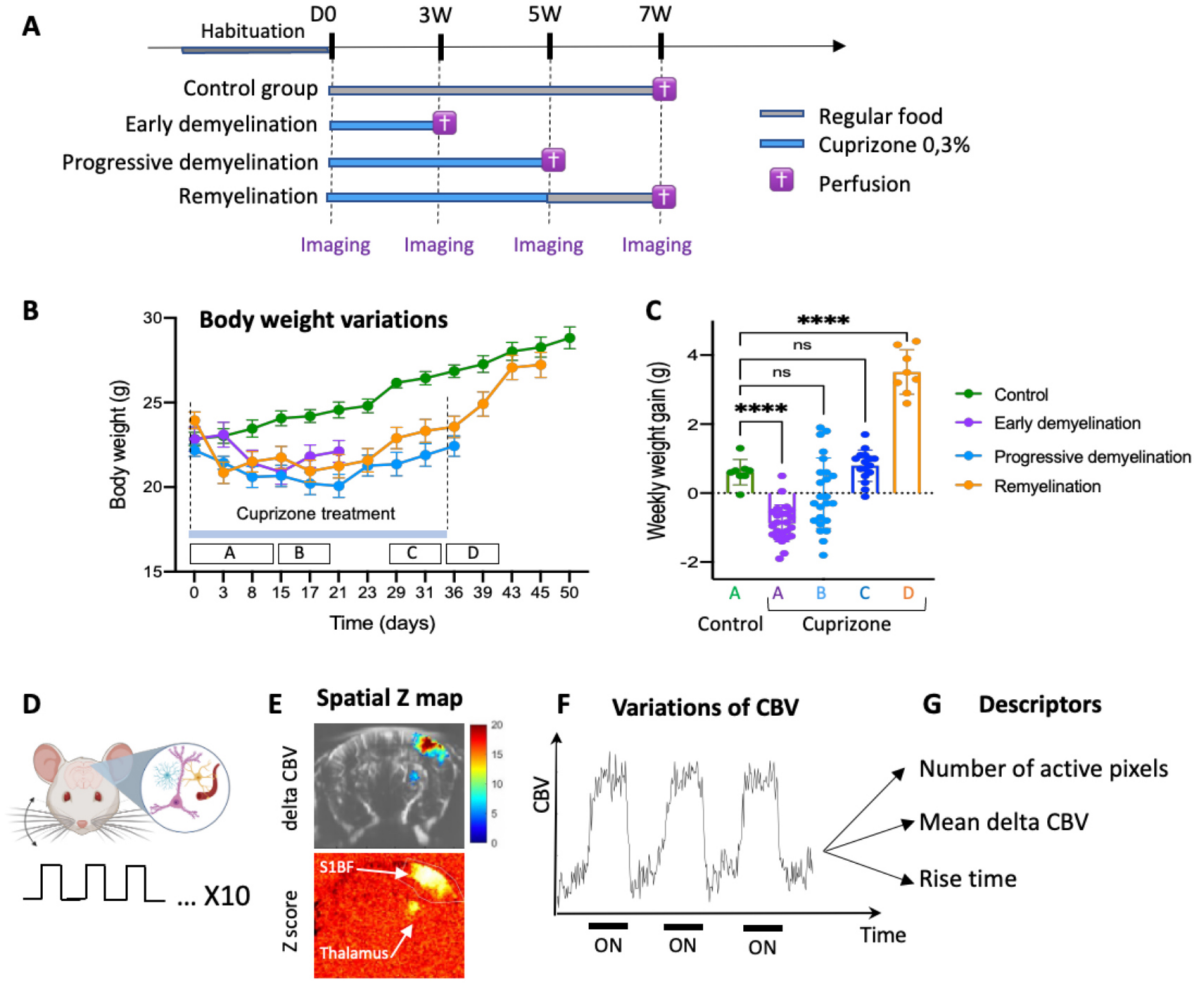
Study of functional hemodynamic response to whisker stimulation in an animal model of central demyelination. (A) Experimental design. The study included four cohorts of 8 mice each. Control group: Fed with regular food throughout the study. Early Demyelination group: Fed with 0.3% cuprizone for 3 weeks. Progressive demyelination group: Fed with 0.3% cuprizone for 5 weeks. Remyelination group: Fed with 0.3% cuprizone for 5 weeks, followed by regular food for 2 weeks. (B, C): Variations of body weight (B) and weekly weight gain (C) in these different cohorts of animals showing the impairment of weight gain induced by cuprizone treatment and its recovery at discontinuation of the treatment. Results are expressed as mean weight (g) or weekly weight gain (g) +/- SEM in N = 8 animals per group. One-way ANOVA, followed by Dunnett’s multiple-comparison test. ****p < 0.0001. In the panels B and C, the letters A–D indicate the time period chosen for the calculations (A: days 0–15 (D0–D15), B: D15–D21, C: D29–D36, D: D36–D43). (D) Functional hyperaemia triggered by repetitive (10X) mechanical whisker stimulation, was evaluated using functional ultrasound (fUS) imaging in the primary sensory cortex barrel field (S1BF). Increased neuronal activation and neurovascular coupling mechanisms result in detectable increases in cerebral blood volume (CBV). (E) Activation Maps and Quantification. Bottom Panel (E): Z-score activation maps show significant regions of increased CBV (Bonferroni-corrected p < 0.05) in the S1BF and thalamus. Top Panel (E): Example of CBV increase in S1BF and thalamus. F. Hemodynamic Response Descriptors. Three descriptors were quantified based on the active pixels in S1BF: Number of Active Pixels, steady-state CBV Variation (ΔCBV) and the rise time. Design Notes: Panels A and D were created usingBioRender.com.

Our experimental design adhered to the ARRIVE guidelines and included 32 adult mice. After a 1-week acclimation period, the animals were randomly assigned to four equal groups (N = 8 each) and underwent baseline imaging (D0). Following this imaging session, each group was treated as follows (Fig. 1A):

- Control group: Received a regular diet throughout the 7-week study. These animals were imaged at 3 W, 5 W, and 7 W post-baseline.
- Early demyelination group: Fed a 0.3% cuprizone diet for 3 weeks and imaged at 3 W before perfusion.
- Progressive demyelination group: Fed a 0.3% cuprizone diet for 5 weeks. Imaging was performed at 3 W and 5 W to follow and monitor the progression of evoked hemodynamic response during demyelination.
- Remyelination group: Fed a 0.3% cuprizone diet for 5 weeks, followed by a return to regular diet for 2 weeks. These animals were imaged at 5 W and 7 W (Fig. 1A).

At the end of the final imaging session, all animals were perfused intracardially to collect brain tissue for Myelin Basic Protein (MBP) immunofluorescent staining (detailed below). No animals were excluded from the study. The experimenter conducting the functional ultrasound (fUS) imaging was blinded to treatment groups.

### 2.4 Cuprizone administration

The experiments used 32 male adult mice (C57BL/6 Rj, 2–3 months old, 20–30 g, from Janvier Labs, France). Mice were housed 8 per cage (also per group) in very large cages (dimensions: 300 mm x 234 mm x 412 mm, Floor space: 916 cm²), under controlled conditions (22 ± 1°C, 60 ± 10% relative humidity, 12/12 h light/dark cycle) with food and water available a*d libitum*. Animals acclimated to housing conditions for 1 week prior to the experiment.

Demyelination was induced in the three treatment groups by feeding the animals with a diet containing 0.3% cuprizone (bis-cyclohexanone oxaldihydrazone; Sigma-Aldrich Inc., St. Louis, MO, USA) for 3 or 5 weeks. Following recommendations (Kipp, 2024;Zhan et al., 2020), 0.3 g of fresh Cuprizone powder was mixed every day with 100 g of ground food (SAFE, France). Mice were weighed twice weekly to monitor weight loss, and all cages were devoid of enrichment.

### 2.5 Myelin immunofluorescent staining and quantification

#### 2.5.1 Perfusion

At the end of the imaging session, while the animals were still anesthetized, they received an intraperitoneal (IP) injection of 0.05 mL of Euthasol (Dechra Pharmaceuticals, 364.60 mg/mL pentobarbital solution). A thoracotomy was performed, and the right atrium was incised. Animals were perfused transcardially with 10 mL of saline solution (0.9% NaCl), followed immediately by 40 mL of 4% paraformaldehyde. Brains were extracted, fixed overnight in 4% paraformaldehyde at 4°C, and cryoprotected in a 30% sucrose solution for 2 days. Brains were frozen in an OCT (Optimal Cutting Temperature) matrix using chilled isopentane (−40°C) on dry ice and stored at -80°C. Brain sections (12 μm thick) were prepared using a cryostat (Leica CM 3050S, Wetzlar, Germany), mounted on Superfrost slides (Thermofisher Scientific, Waltham, MA, USA), and stored at -20°C in cryoprotectant until processing for all animals was complete.

#### 2.5.2 Myelin-Basic Protein (MBP) immunohistochemistry

Two slides per animal (each one containing eight sections) were washed three times in 0.1 M phosphate-buffered saline (PBS) with 0.9% NaCl. Sections were incubated overnight at room temperature with the primary antibody (mouse anti-MBP, clone 12, Chemicon, Avantor-VWR, #MAB384, dilution: 1:150, diluted in 0.3% triton X-100). After the three additional PBS washes, sections were incubated for 2 h with the secondary antibody (Alexa Fluor 488-conjugated donkey anti-mouse antibody, dilution: 1:1000; Invitrogen). Finally, sections were cover slipped using Fluoromount (Sigma-Aldrich). All staining was performed simultaneously for all animals to ensure consistency.

#### 2.5.3 Microscopic observation and quantification

As this model induces a widespread but heterogenous demyelination of the brain, we chose to quantify MBP immunofluorescent staining in several areas involved in sensory processing, including the primary sensory cortex (barrel field), the thalamus (VPM, VPL), the internal capsule, the medial corpus callosum, and the hippocampus.

**Fig. 2.**
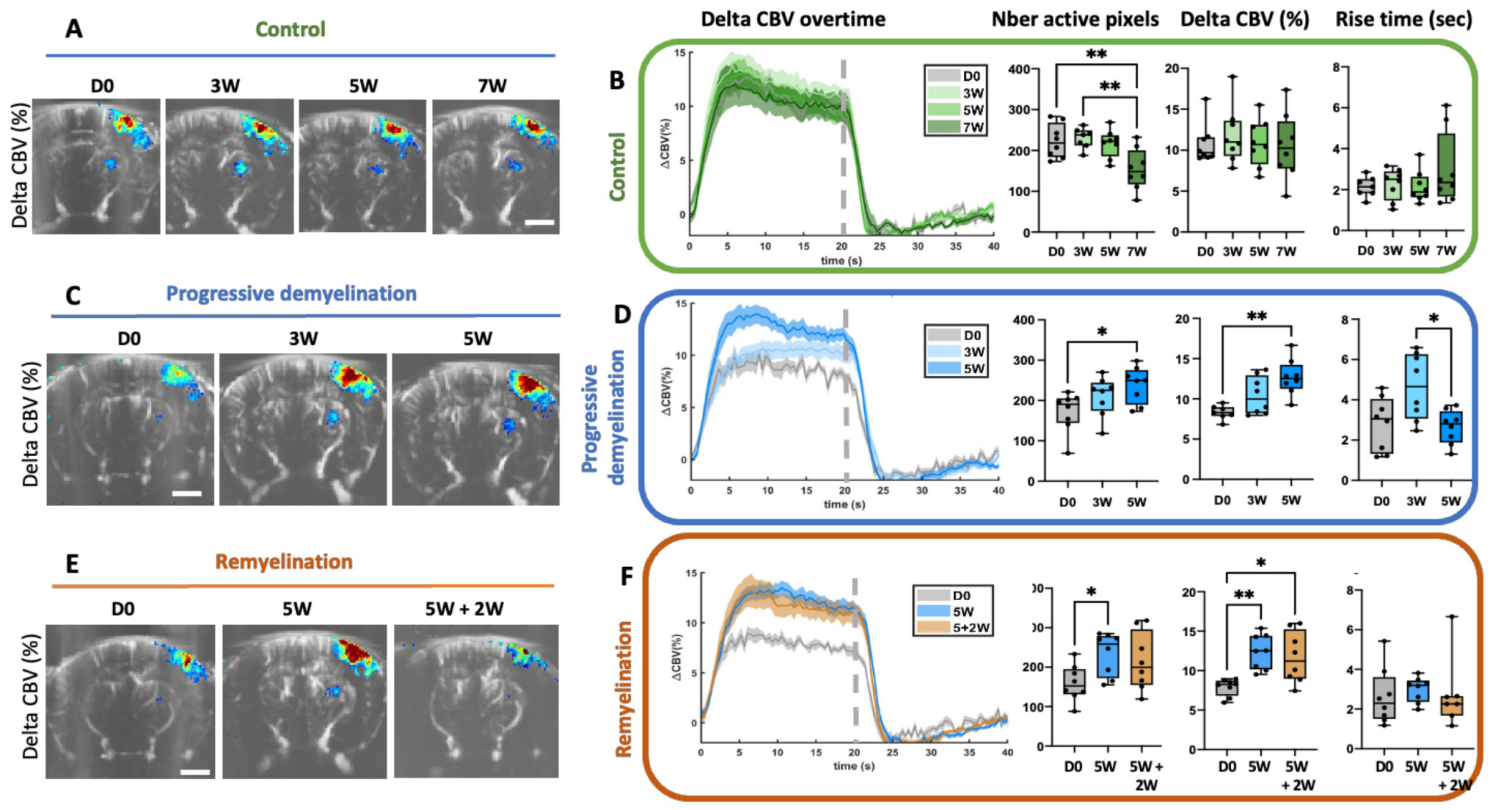
Longitudinal follow-up of the cortical hemodynamic response during demyelination induced by Cuprizone intoxication and its spontaneous (partial) remyelination. Panels A, C, and E present representative spatial maps of CBV changes evoked by whisker stimulation in: (A) a control animal at different time points of the study, (C) a mouse before (D0) and at two time points during progressive demyelination (3 W and 5 W), and (E) a mouse imaged before treatment (D0), after 5 weeks of demyelination (5 W) and after 2 weeks of partial spontaneous remyelination (5 W + 2 W). Scale bars in A, C, and E: 1 mm. Panels B, D, and F display the quantification of the relative CBV changes evoked by whisker stimulation (n = 8 per group) for three cohorts of animals: (B) control animals over time, (D) animals of the ‘progressive demyelination’ treated with cuprizone for 5 weeks and imaged at different time points, and (F) animals from the ‘Remyelination’ group, treated with cuprizone for 5 weeks (blue), followed by 2 weeks of remyelination (orange). The curves in transparency define the SEM. Each panel (from left to right) within B, D, and F depicts: i) the mean time-course of ∆CBV induced by whisker stimulation. The dotted lines mark the end of the stimulation period (20 sec stimulation), ii) The number of active pixels, iii) The steady-state CBV variation (∆CBV, expressed as a percentage within the 7–20 sec stimulation interval), and iv) the rise time. Data are presented as mean ± SEM with individual values overlaid. Since the data reflect measurements from individual animals followed longitudinally, a repeated-measures one-way ANOVA was used for statistical analysis, followed by Tukey’s test for post-hoc multiple comparisons. For clarity, only statistically significant comparisons are shown in the figure. *p < 0.05, **p < 0.001.

All stained sections were digitized using a nanozoomer (Zeiss, facility of the Vision Institute, Paris) uniform acquisition parameters. The resulting files were opened with the manufacturer’s software, and two sections per animal, corresponding to coordinates: Bregma -1.34 and -1.70 mm were exported in PNG format. These files were then opened with the Fiji software (ImageJ.net) and transformed into 256- level grayscale images. Two types of quantification were performed:

i) Measurement of the mean gray intensity
  Since our immunostainings were performed using indirect immunofluorescence, with all sections stained simultaneously, the measurement of mean gray intensity serves as a direct and linear indicator of the amount of MBP present in the tissue. For each image, the mean gray value of the background (outside the brain; seeSupplementary Fig. 2a) and the MBP staining in various brain regions (Supplementary Fig. 2b–f) were quantified on both the right and left sides. After subtracting the background level for each section and averaging the values from the right and left sides, the data from the two sections were further averaged. The analysis was conducted by an experimenter blinded to the treatment groups. Results were expressed as mean gray intensity ± SEM.
ii) Measurement of the percentage of area covered by the staining
iii) Using the same 8-bit black-and-white images, we performed an additional quantification of the percentage of the area covered by staining. A common threshold value (64) was applied to all sections using ImageJ. The area occupied by the staining was then measured for both the right and left sides and averaged across two sections per animal. Results (presented inSupplementary Fig. 5) were expressed as the mean percentage area ± SEM. This quantification method yielded results consistent with those obtained using mean gray intensity, with a strong linear regression coefficient in the S1BF region for all animals (R² = 0.701).

### 2.6 Imaging functional hyperaemia and quantification

#### 2.6.1 Anesthesia

The mice were initially anesthetized with 2% Isoflurane and an intramuscular (IM) injection of medetomidine (Domitor, 0.08 mg/kg). Anesthesia was maintained using a continuous IM injection of medetomidine (0.08 mg/kg/h) delivered via a syringe pump, in combination with 0.5% isoflurane (0% O_2_, 100% air as carrier gas). Anesthesia depth was monitored throughout the imaging sessions using the cardiac and respiratory frequency measurements (Labchart). To prevent hypothermia, body temperature was maintained at 37°C with a rectal probe connected to a heating pad. To protect the mice’s eyes, an ophthalmic ointment (Ocry-gel, TVM, UK) was applied.

Skin alterations, such as scars or local inflammation of the skin above the skull, can reduce the fUS signal, due to ultrasonic wave reflections. To remove such potential artifacts, skin above the imaging site was surgically prepared at the start of each imaging session under anesthesia. Following a local subcutaneous injection of Lidocaine. the skin was incised and opened below the ultrasound probe. Sterile ultrasound gel (1 mL) was applied to the exposed skull. At the end of the imaging session, the skin was sutured with 6.0 sutures (Ethilon, Phymep, France).

#### 2.6.2 Functional ultrasound (fUS) imaging

To enhance reproducibility in probe placement, the imaging plane (Bregma -1.60 mm) was determined using Icostudio software (Iconeus, Paris, France). A linear scan (6 mm span along the anterior-posterior axis, 0.2 mm step size) was conducted at the beginning of each session, co-registered to an average scan, and aligned with a standard Doppler reference template pre-aligned to the Allen Mouse Brain Atlas common coordinate framework (Nouhoum et al., 2021). This method allowed reproducible placement of the probe above the same imaging plane (coronal plane of the S1BF) with a reproducibility of 200 μm along both X and Y axes (Nouhoum et al., 2021).

Imaging was performed using the Iconeus One scanner (Iconeus, Paris, France) designed for small animals. The linear probe, consisting of 128 piezoelectric transducers with a 15 MHz central frequency, acquired ultrafast images at 500 Hz using the coherent summation of 11 compounded tilted plane waves emitted at a 5,500 Hz pulse repetition frequency.

#### 2.6.3 Whisker stimulation

To standardize whisker stimulation, we used an Arduino Uno-controlled (https://store.arduino.cc/;Bertolo et al., 2021). This setup synchronized stimulation with data acquisition. The servomotor moved a cotton bud perpendicular to the whiskers at 4 Hz. After a 20-sec baseline period, stimulation was applied for 20 sec, followed by 20 sec of rest, repeated 10 times. Total acquisition time was 420 sec.

#### 2.6.4 Signal analysis

Blood signal was separated from tissue signal using a singular value decomposition (SVD) clutter filter. Each power Doppler frame represented the average of 200 filtered ultrafast frames; resulting in a 2.5 Hz frame rate. Following established methods (Demené et al., 2015), a clutter filter removed the tissue signal by eliminating the 60 first singular vectors that correspond mainly to the tissue space. The filtered frames were integrated to produce a Power Doppler image every 400 msec. No motion correction was applied.

A detrending step removed slow signal drift during acquisition. Activation maps displaying significantly active areas (Z scores with p < 0.0000006 after Bonferroni correction) were computed using a general linear model (GLM) analysis in Matlab (Fig.1E, bottom panel). The spatial extent of functional hyperemia was quantified by calculating the number of significantly active pixels. Temporal signal data from active pixels were fitted using MATLAB’s “stepinfo” function, which computed parameters such as the rise time (Supplementary Fig. 1).

#### 2.6.5 Statistical analysis

All statistical analyses were performed using GraphPad Prism 10.

Data normality was assessed using Shapiro-Wilk’s test, and homoscedasticity was evaluated with Barlett’s test.

Statistical comparisons of mean gray density for MBP immunostaining across brain regions were conducted using:

1. ANOVA with Tukey’s multiple-comparison test: For normally distributed data with homoscedasticity.
2. Brown-Forsythe and Welch ANOVA with Dunnett’s T3 test: For normally distributed but heteroscedastic data.

Hemodynamic descriptors (number of active pixels, rise time, and ∆CBV) were analyzed using repeated-measures one-way ANOVA, followed by Tukey’s post- test.

### 2.7 Correlation using multiple linear regression

As the development of new pharmacological treatments requires a longitudinal measure of the level of myelin loss, this study aimed at investigating the use of longitudinal measurement of the altered hemodynamic response as a surrogate for the level of myelin. To do so, we investigated the potential ability of the three descriptors of the hemodynamic response measured by fUS imaging (number of active pixels, steady-state CBV variation, and the rise time) to predict the level of myelin (quantified by MBP immunohistochemistry at the end of the experiment). To do so, we trained a multiple linear regression model (consisting in a successive and automatic removal of the less important predictors) with the three descriptors as inputs, using the Matlab function ‘stepwise’. To assess the overall performance of the model, we conducted an F-test to compare the model with all its predictors to a constant model (analysis of variance test). Furthermore, we assessed the contribution of each predictor by testing whether the corresponding parameter significantly differs from zero using the classic t-test.

### 2.8 Role of the funding source

The funding source had no role in data collection, analysis, interpretation, the writing of the manuscript, or to submit the manuscript for publication.

## 3 Results

### 3.1 Induction of myelin loss by Cuprizone treatment

While in control animals the average weekly weight gain remained relatively constant at 0.61 g ± 0.13, in contrast, cuprizone treatment significantly impacted weight gain, though this effect varied over time.

From days 3 to 15, animals on cuprizone consistently lost weight, with an average loss of -0.88 g ± 0.19 (Fig. 1B, C). Between days 15 and 21, there was heterogeneity in weight changes: while some animals continued to lose weight, others began to gain weight. On average, the change in weight during this period was negligible (0.0 g ± 0.36 g over N = 24,Fig. 1C). During the final week of cuprizone treatment (days 28–35), animals’ weekly weight gain returned to levels comparable to controls, with an average gain of +0.79 g ± 0.16 (Fig. 1C). Notably, reintroducing standard food after cuprizone treatment resulted in a markedly elevated and statistically significant rate of weight gain (+3.5 g ± 0.23). These results are consistent with prior studies (Leopold et al., 2019) and confirm that the cuprizone model impacts the animals’ natural ability to gain weight.

### 3.2 Evoked hemodynamic response induced by whisker stimulations in the primary sensory cortex and thalamus at various stages of demyelination in the cuprizone model

Using functional ultrasound imaging, we quantified potential alterations of the hemodynamic response associated with the progression of demyelination and remyelination (5 weeks cuprizone followed by 2 weeks of recovery). To achieve this, we analyzed three descriptors of the brain’s hemodynamic response to stop mechanical whisker stimulation: the number of active pixels, the steady state CBV variation, and the rise time (Fig. 1D, G;Supplementary Fig. 1).

In the control cohort, we evaluated the stability and reproducibility of these measurements over time in naïve untreated mice. Both steady-state CBV variation and rise time remained consistent across all time points (Fig. 2A, B). However, the number of active pixels was stable for the three first time points (DO, 3 W, 5 W), but significantly decreased at the final time point (7 weeks,Fig. 2A, B). This decline in response over time in healthy mice has been reported previously by other researchers (https://doi.org/10.1101/2023.12.21.572595).

To investigate alterations in functional hyperemia during demyelination, we imaged a second cohort (‘progressive demyelination’) at baseline (D0), at early time point of demyelination (3 W), and at maximal demyelination (5 W). Our findings revealed a progressive increase in the number of active pixels (p = 0.03,Fig. 2C, D), indicating a more widespread activation, along with a gradual rise in steady-state CBV within these pixels (Fig. 2C, D). These effects were statistically significant at 5 weeks of cuprizone treatment. Additionally, the rise time was statistically increased at 3 weeks of treatment, suggesting early alterations in neurovascular coupling dynamics.

The effects of remyelinating on the hemodynamic response were investigated on a third cohort of animals (‘Remyelination’), imaged at baseline, at the peak of demyelination (5 W), and after 2 weeks of spontaneous remyelination (following a return to normal food for 2 weeks, ‘5 + 2W’,Fig. 2E). The effects observed after 5 weeks of cuprizone treatment were consistent with those seen in the previous cohort, confirming the reproducibility of our findings across batches of cuprizone-treated animals. Following remyelination, there was a modest reduction in both the number of active pixels and delta CBV (Fig. 2F). However, this effect showed considerable variability between animals, resulting in more dispersed measures (Fig. 2F). As discussed later, this variability in hemodynamic responses is likely attributable to inter-individual differences in the extent of spontaneous remyelination, a phenomenon previously highlighted byStidworthy et al. (2003).

**Fig. 3.**
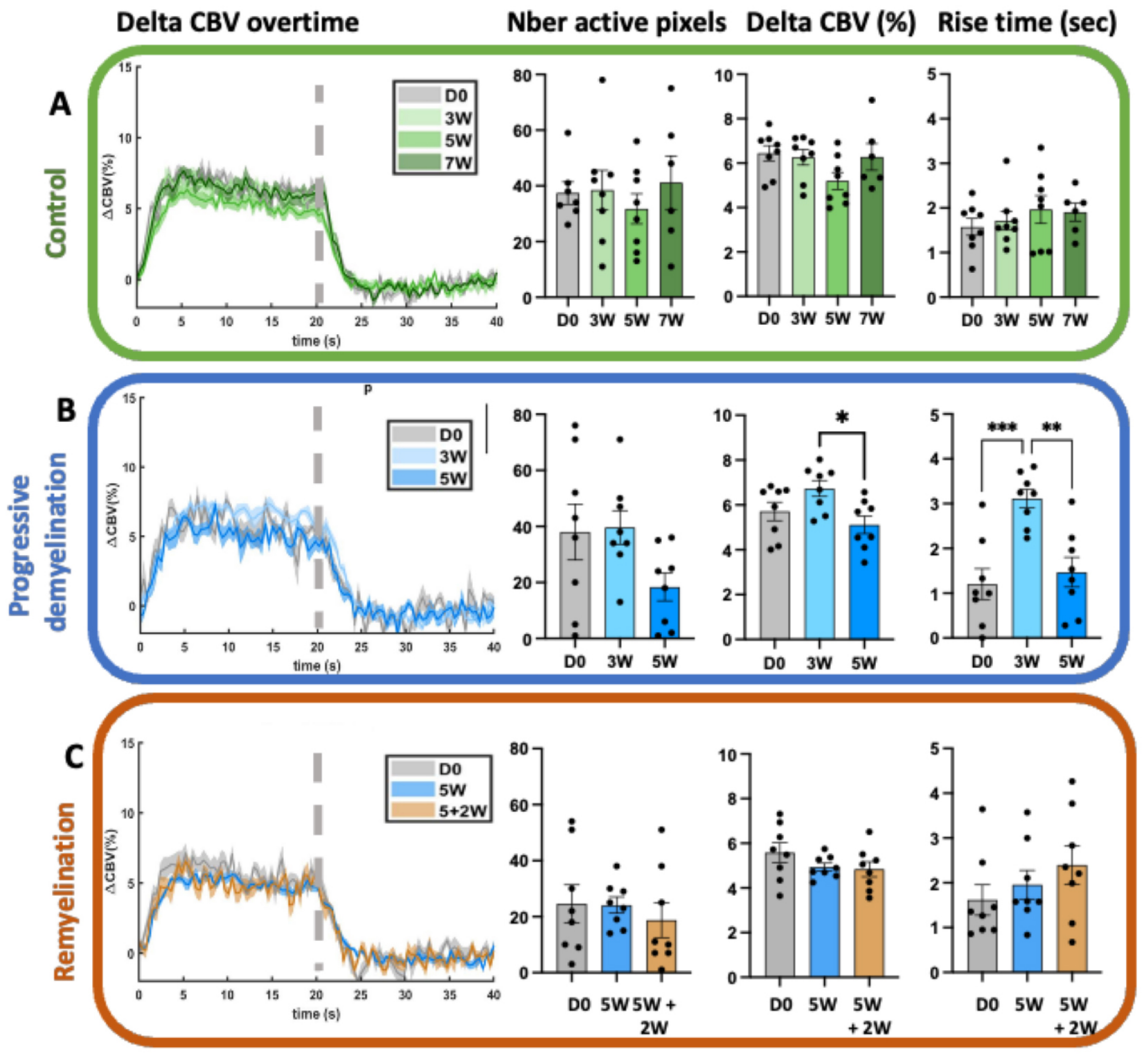
Longitudinal follow-up of the thalamic hemodynamic response during demyelination induced by Cuprizone intoxication and its spontaneous (partial) remyelination. Similar toFigure 2, this figure illustrates changes in the thalamic hemodynamic response evoked by whisker stimulation (n = 8 per group) in three cohorts of animals: (A) control animals observed over time, (B) animals from the ‘progressive demyelination’ group treated with cuprizone for 5 weeks and imaged at different time points, and (C) animals from the ‘Remyelination’ group, treated with cuprizone for 5 weeks (blue) followed by 2 weeks of remyelination (orange). Each set of panels presents: i) the mean time-course of ∆CBV induced by whisker stimulation, ii) The number of active pixels, iii) The steady-state CBV variation (∆CBV, expressed as a percentage within the 7–20-sec stimulation interval), and (iv) the rise time. In all panels, the data are presented as mean ± SEM with individual values overlaid. *p < 0.05, **p < 0.001. For clarity, only statistically significant comparisons are shown in the figure.

To determine whether the observed changes were specific to the cortical level, we investigated whether similar effects occurred in the thalamic relay, specifically in the ventro-posterior-lateral and -posterior-medial thalamic nuclei (VPL, VPM). Notably, changes were evident only during the early demyelination period (3 W), when both the average ∆CBV and rise time increased (Fig 3). By 5 weeks of cuprizone intoxication, the hemodynamic response in the thalamus remained unaffected. This suggests that the enhanced cortical response observed at this stage, coinciding with complete demyelination, may represent a unique adaptation or dysfunction specific to cortical circuits.

Finally, to determine whether small demyelinating lesions in the grey or white matter would induce similar alterations, we performed stereotaxic injections of lysolecithin (LPC) either in the S1BF or the internal capsule, with saline injections in the internal capsule serving as controls. Surprisingly, despite the strong and localized demyelination induced by LPC injections (Supplementary Fig. 3A), the evoked hemodynamic response remained unchanged compared to saline-injected animals (Supplementary Fig. 3C).

### 3.3 Quantification of the level of demyelination in various brain areas

**Fig. 4.**
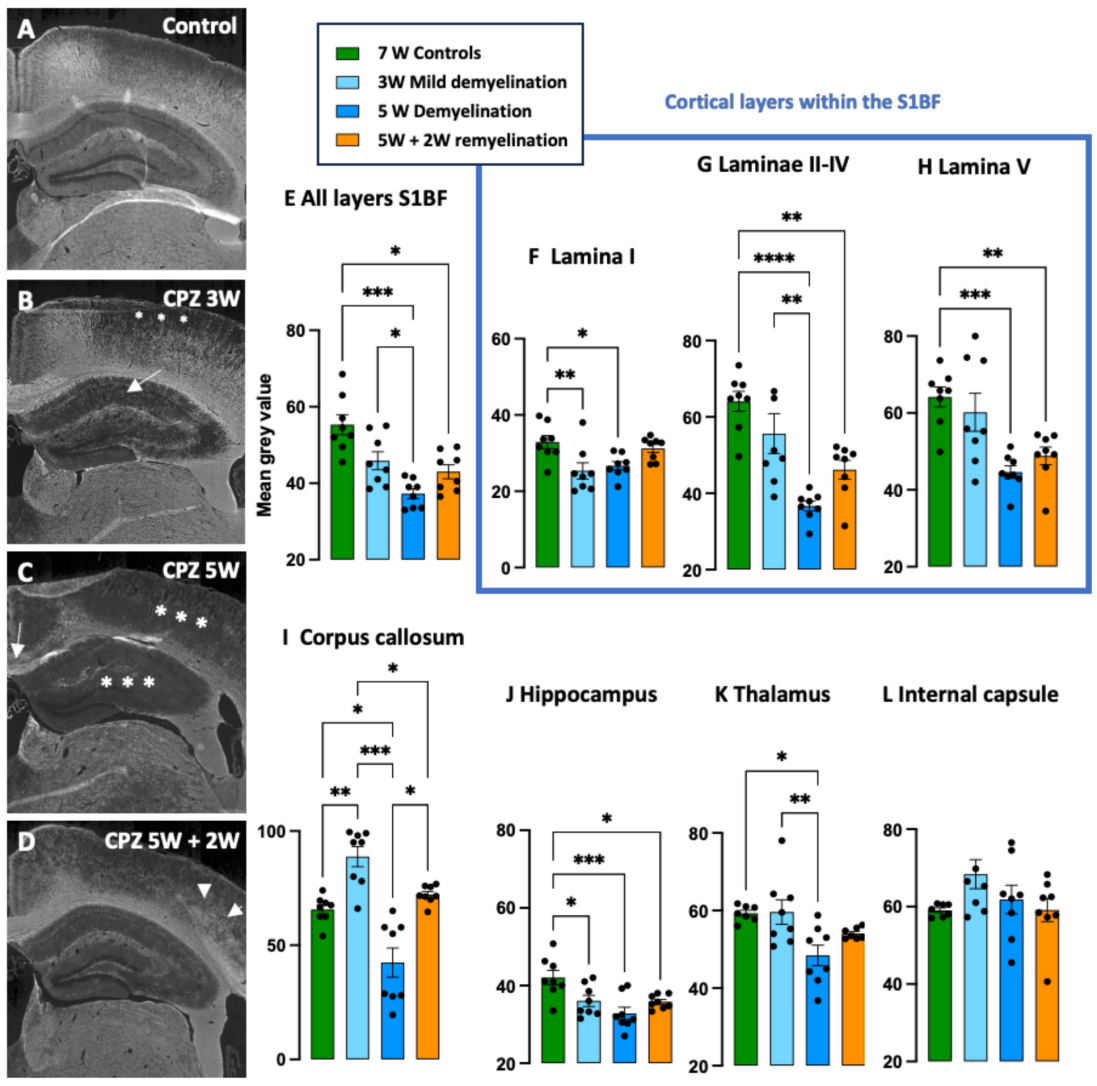
Quantification of the level of relative level of myelin by immunofluorescent staining of the Myelin Basic Protein. At the end of the imaging protocol, all animals were anesthetized, perfused, and their brain was sectioned for MBP immunofluorescent staining, which was performed simultaneously in all animals. The fluorescent staining was scanned automatically using a nanozoomer, with identical acquisition settings applied across all samples. Panels A–D show representative examples of the MBP immunostaining in the S1BF cortex, corpus callosum, and hippocampus for a control animal (A), an animal intoxicated with Cuprizone (CPZ) for 3 W (B), 5 W (C), and an animal allowed to remyelinate for 2 weeks following 5 weeks of cuprizone intoxication (D). In panel B, the stars illustrate the initial stages of demyelination in the superficial cortical layers, while the arrow indicates the demyelination in the hippocampus. In panel C, the arrow highlights strong demyelination in the corpus callosum (arrow), while the stars denote cortical demyelination. In panel D, arrows indicate patches of remyelination in the S1BF. Scale bar: 1 mm. Panels E–L present the quantified MBP immunostaining, showing the loss of myelin induced by CPZ treatment in the S1BF (E–H), medial corpus callosum (I), hippocampus (J), and thalamus (K) and the extent of complete or partial remyelination depending on the brain region. At the studied time point, no statistically significant demyelination was observed in the internal capsule (L). Results are displayed as mean ± SEM with individual values overlaid. Statistical analyses were performed suing ANOVA followed by Tukey’s multiple-comparison test for data with normal distribution and homoscedasticity. For data that were normally distributed but heteroscedastic (S1BF, hippocampus and internal capsule), the Brown Forsythe and Welch ANOVA tests were applied, followed by Dunnett’s T3 multiple-comparison tests. For clarity, only statistically significant comparisons are shown in the figure. *p < 0.05, **p < 0.01, ***p < 0.001. NS: Non-significant. N = 8 per group.

The level of demyelination was assessed using MBP immunofluorescent staining in several areas of the brain. Two approaches of MBP quantification were compared, yielding consistent results (Fig. 4;Supplementary Fig. 4). After 3 weeks of cuprizone treatment, a stage widely described in the literature as an early phase of demyelination process in this model (Kipp et al., 2017;Matsushima & Morell, 2006;Vega-Riquer et al., 2019), we observed a strong and statistically robust demyelination in both the primary sensory cortex barrel field region (S1BF) and the hippocampus (Figs. 4A, B, Eand4J). Within the cortex, the lamina I exhibited the most pronounced demyelination of all layers (Fig. 4F). Interestingly, at this time point, an unexpected increased MBP immunostaining was observed in the corpus callosum (Fig. 4I).

As anticipated, 5 weeks of cuprizone treatment led to extensive demyelination across several brain regions, including the S1BF (Fig. 4C, E), the medial corpus callosum (Figs 3E and 4C), the hippocampus (Fig. 4C, J), and the thalamus (Fig. 4K). In the cortex, the laminae II-III and V showed severe demyelination (Fig. 4G, H). Transitioning to normal food for 2 weeks induced remyelination in the four aforementioned areas (Fig. 4D–K). Similar results were obtained using an alternative method of MBP quantification (Supplementary Fig. 5).

Interestingly, despite some inter-animal variability of MBP expression across the experimental groups, we could not establish demyelination or remyelination in the internal capsule (Fig. 4L).

### 3.4 Correlation of the level of demyelination with descriptors of the hemodynamic response in fUS imaging

To investigate potential direct linear correlation between changes of hemodynamic response and the level of demyelination in the brain of cuprizone-treated animals, we studied and first analyzed the statistical correlations between the three descriptors of the hemodynamic response and the MBP immunostaining. Individual values from all animals included in the study were used. Results are presented inFigure 5A.

**Fig. 5.**
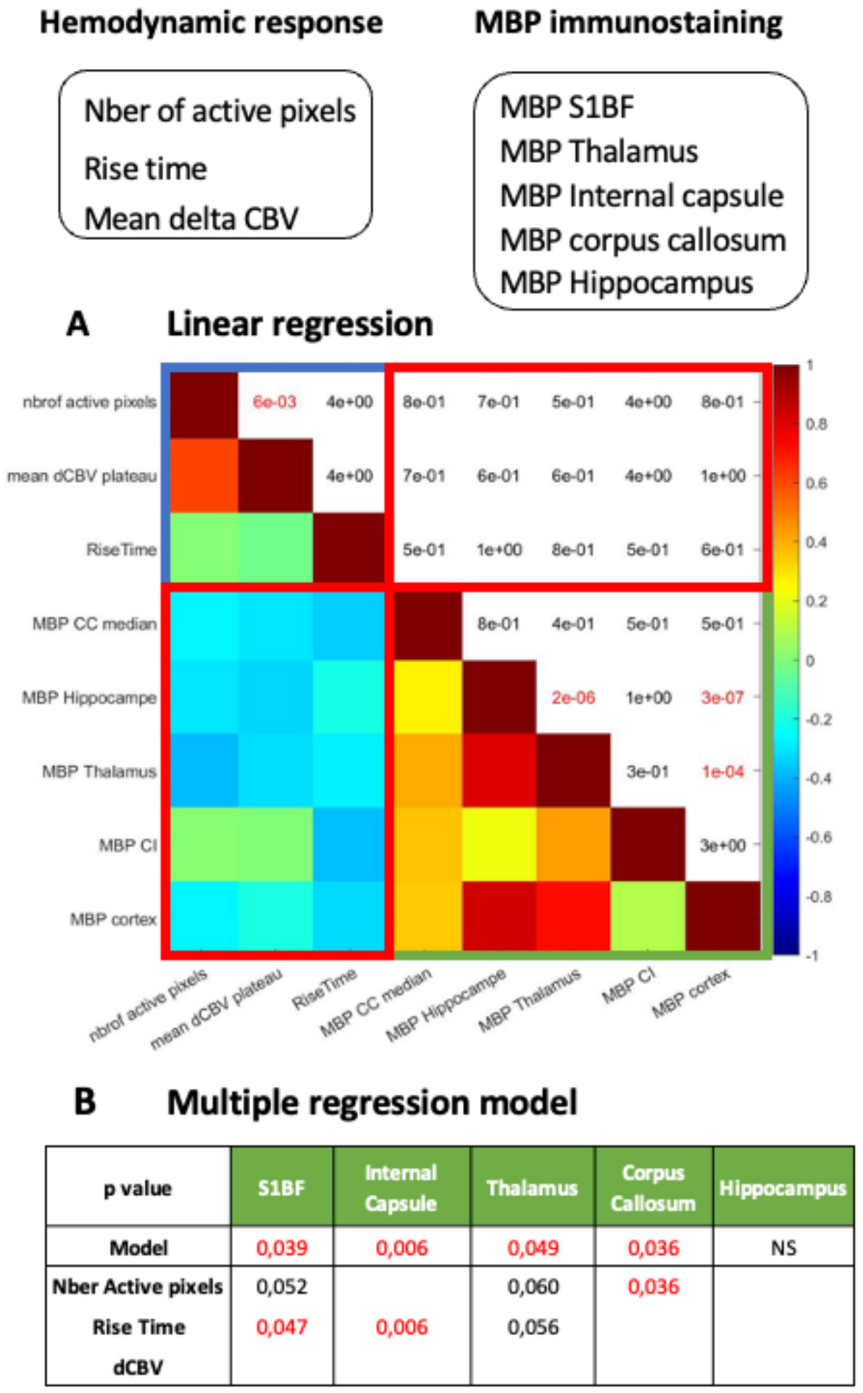
Correlation of the three descriptors of the evoked hemodynamic response (alone (A) or in combination (B)) with the individual level of MBP. (A) The correlation between the altered hemodynamic response and MBP expression levels was analyzed using linear regression, based on individual values. Results are presented as a spearman correlation matrix. The half left of the matrix shows the correlation coefficients, where positive correlations are represented in red, and negative correlations in blue. The top-right half matrix displays the p values, indicating the statistical significance of the correlations. These p-values were corrected for multiple comparisons using the Benjamini-Hochberg method. Statistically significant correlations (highlighted in red) were observed only between specific fUS measures and within changes in MBP expression in three brain regions. (B) To further explore potential associations between the descriptors extracted from the evoked hemodynamic response and the level of myelin of individual animals across various brain regions, multiple regression models were fitted. The table reports the p-values (corresponding to the null hypothesis that the estimate is equal to zero) of the models and descriptors for the model trained. The results indicate that the rise time correlates well (alone or in combination with other descriptors) with the MBP content in the S1BF and the internal capsule. Additionally, one model identified one association between the extent of MBP loss in the medial corpus callosum and the number of active pixels.

In the correlation matrix, positive correlation (yellow/red color coded) and anticorrelation between these metrics (green/blue color code) are shown in the bottom left, while the p-values from the Spearman correlation test (corrected for multiple test comparisons) are displayed on the top right of the matrix. Statistically significant correlations (p-value < 0.05) are indicated in red.

Interestingly, the number of active pixels was strongly and positively correlated with the mean CBV plateau (p-value = 6 10^-3^,Fig. 5A). Additionally, the extent of MBP loss in various brain areas demonstrated a strong positive correlation between the MBP expression level in the hippocampus and the thalamus. Similarly, MBP levels in the cortex were strongly and significantly correlated with those in the thalamus (p = 10^-4^) and hippocampus (p = 3 10^-7^,Fig. 5A).

Overall, changes in MBP expression were anticorrelated with the descriptors of the hemodynamic response, but these anticorrelations were not statistically significant.

To assess the predictive capacity of the hemodynamic response descriptors for determining myelin levels in five brain regions, we used linear regression models (Fig. 5B). For each brain region where MBP was quantified, a linear model was trained using the three descriptors selected through stepwise regression. This process involved the successive removal of the least significant predictors, performed automatically using the MATLAB ‘stepwise’ function.

As shown inFigure 5C, the models indicate associations between the MBP levels and hemodynamic descriptors in all regions except the hippocampus. To identify the most significant predictors, we examined the estimates and corresponding p-value for each predictor in the model (bottom row in the table,Fig. 5B).

This analysis revealed the following: i) The number of active pixels and the rise time together correlated with the MBP content in the S1BF and the thalamus, with the effect being more pronounced in the S1BF and primarily driven by the rise time. ii) The rise time alone correlated with the MBP immunofluorescent level of MBP in the internal capsule. iii) The number of active pixels alone was linked to MBP levels in the corpus callosum.

Notably, the rise estimate was consistently negative across all models (-2.26 for the internal capsule, -1.38 for the thalamus, and -1.57 for the S1BF). This was expected, as myelin loss delays the neural signal, resulting in an increased rise time.

## 4 Discussion

Given the urgent need for non-invasive methods to measure pathophysiological changes in preclinical models of demyelination and the scarcity of studies investigating evoked cerebral hemodynamic responses—often altered in clinical observations—our study leveraged the high sensitivity of fUS imaging to study whisker stimulus-evoked hyperemia in the cuprizone model in mice. Our study not only underscores the potential of using altered hemodynamic responses as a correlate for myelin loss in this model, but also reveals the importance of vascular health in the progression of demyelinating diseases.

### 4.1 Cuprizone induced inhomogeneous brain demyelination

To assess the extent of myelin loss across brain regions, we quantified Myelin Basic Protein (MBP) via immunofluorescent staining, a surrogate for myelin content. More accurate techniques like electron microscopy or spectral confocal reflectance imaging (Gonsalvez et al., 2019) exist, but unfortunately they could not be used within the framework of this study. MBP staining still yielded valuable insights. Most studies focus on the medial corpus callosum (Gudi et al., 2009;Matsushima & Morell, 2006;Vega-Riquer et al., 2019), but we observed widespread demyelination with variability between regions (Goldberg et al., 2015). The superficial cortical laminae and the hippocampus showed MBP loss after just 3 weeks, while the thalamus and cortical layers II-V were affected only after 5 weeks, consistent with prior research (Goldberg et al., 2015;Gudi et al., 2009;Norkute et al., 2009;Silvestroff et al., 2010;Skripuletz et al., 2008;Yang et al., 2009). Remyelination patterns also varied, with complete recovery in the corpus callosum but incomplete and inconsistent recovery in the cortex, thalamus, and hippocampus.

As sensory afferents from the whiskers go through the trigeminal SpV nucleus to the thalamic relay (Wimmer et al., 2010), and then the internal capsule to finally contact cortical neurons (Petersen, 2007), demyelination at any point along this pathway could underlie the delayed hemodynamic response seen in cuprizone-treated animals. Indeed, our results confirm that the abnormal hemodynamic response in the S1BF linked with the loss of MBP in these three neuroanatomical structures (thalamus, internal capsule, and S1BF). Interestingly, the descriptor significantly linked with the MBP alteration in these structures was the rise time. This increased rise time suggests a delay in the hemodynamic response and relates to the latency of evoked potentials, which is a measure classically used in the diagnosis of MS (Compston & Coles, 2008) and was previously demonstrated to be a preclinical marker of remyelination in pre-clinical models of MS (Cordano et al., 2022;Marenna et al., 2022). Both the increased rise time and evoked potentials latency are due to myelin damage and the subsequent reduction in neuronal conduction.

Notably, cuprizone induced only modest alterations in the thalamic hemodynamic response. One observed change—an increased rise time at the early stage of treatment—mirrored that seen in the cortex. Given the thalamus’s upstream position relative to the cortex, the delayed cortical response may therefore stem from delays originating in the thalamic relay.

### 4.2 Clinical relevance of the increased evoked hemodynamic response observed in the cuprizone model

The cuprizone model, widely used to study demyelination (Vega-Riquer et al., 2019), induces chronic, non-immune-mediated demyelination of the brain through oligodendrocyte degeneration. To explore changes in evoked response across different phases of demyelination or remyelination, we conducted a longitudinal study where each animal serves as its own control. Our findings reveal that after 5 weeks of cuprizone treatment, the whisker stimulus-evoked hyperemia was consistently increased. This was characterized by a larger activated area (more active pixels) and enhanced activation within this region (greater CBV over time).

These findings are highly relevant to the clinical condition (Rocca et al., 2005), as in MS patients with clinically isolated syndrome (CIS;Rico et al., 2011;Rocca et al., 2003), with non-disabling (Rocca et al., 2002) or mildly disabling relapsing-remitting (RR) MS (Reddy et al., 2002), exhibit increased activation of the primary sensorimotor cortex during motor tasks. These enhanced responses (see for reviewRocca et al., 2022) have been considered to play a compensatory role in RRMS, since it was related to maintaining a good task performance despite the presence of widespread structural damage, and are believed to be due to both altered cerebral blood flow and oxygen consumption (Hubbard et al., 2017;West et al., 2021).

This interesting parallel between our results and the clinical condition suggests that even though the cuprizone model does not model all hallmarks of the clinical condition, it modeled well its altered hemodynamic responsiveness.

Several authors have hypothesized that these surprising, enhanced responses might be an adaptive plasticity to compensate for the growing disability of the diseased brain. The cellular and molecular mechanisms behind this plasticity are still largely unknown. However, a large body of literature describes an alteration of the vascular system and of the neurovascular unit in MS (Caprio & Russo, 2016), opening the debate on whether vascular events may be the primary cause of neurological diseases or rather a mere participant recruited from a primary neuronal origin. Our findings suggest that demyelination itself, caused by oligodendrocyte disruption, can promote neurovascular coupling alterations. Importantly, the absence of hemodynamic changes in models with localized gray or white matter demyelination (e.g., LPC-induced lesions) indicates that local neuroinflammation and demyelination alone are insufficient to induce these changes. Given that oligodendrocyte disruption is a hallmark of the cuprizone model, it is plausible that these vascular dysfunctions stem from oligodendrocytic origins. Further research is required to validate this hypothesis.

One of the limitations of our study is the lack of investigation of the extent of this neurovascular alteration at later stages (10–12 weeks of cuprizone treatment), which mimic aspects of advanced MS characterized by axonal damage. Examining functional responses at these time points could determine whether, as in MS patients, responses decrease during progressive disease stages. Future work will focus on this critical question.

### 4.3 Measure of vascular dysfunction in Cuprizone-induced demyelination

Interestingly, the amplitude of the response (CBV) alone was not associated with the extent of MBP staining, either independently or in combination with other factors. Given that these changes closely mimic those observed in the early phases of multiple sclerosis (MS) (Rocca et al., 2022), we hypothesize that this measurement is a critical indicator of the vascular dysfunction inherent to the model. As highlighted by various studies (Caprio & Russo, 2016;Cashion et al., 2023;Sweeney et al., 2018), MS patients frequently experience vascular complications, such as blood-brain barrier leakage (Sweeney et al., 2019), microbleeds, and reduced cerebral blood flow. A growing body of evidence suggests that the dysfunction of various cells within the neurovascular unit—such as endothelial cells (Zlokovic, 2008), astrocytes (Correale & Farez, 2015), pericytes (Zhang et al., 2020), and microglial cells (Lassmann et al., 2012)—plays a significant role in MS pathology (Ballabh et al., 2004;Cashion et al., 2023;Sweeney et al., 2019). Therefore, targeting the stabilization of the vasculature by blocking the pathological changes affecting these cells represents a promising therapeutic avenue in MS (see review byCashion et al., 2023).

In conclusion, our study highlights the importance of vascular health in the progression of demyelinating diseases. Future research should focus on further characterizing these vascular dysfunctions over longer time frames and exploring therapeutic strategies aimed at preserving neurovascular integrity. By advancing our understanding of the interplay between demyelination and vascular dysfunction, we hope to contribute to the development of more effective diagnostic tools and treatments for MS and related neurological conditions.

## Supplementary Material

Supplementary Material

## Data Availability

Source data are available on the repository website zenodo using the following link:https://zenodo.org/records/13136108. Custom codes used for the analysis of fUS data used in this study are protected by INSERM. The rest of this work did not involve any other particular code.
