# Supplementary material for: Novel insights into vascular dysfunction in cuprizone-induced demyelination through functional ultrasound imaging
Source: Imaging Neurosci (Camb). 2025 Apr 10;3:imag_a_00534. doi: 10.1162/imag_a_00534 (PMC12319804; doi:10.1162/imag_a_00534)
Supplement: Supplementary Material [file imag_a_00534-supp.pdf]

Supplementary materials

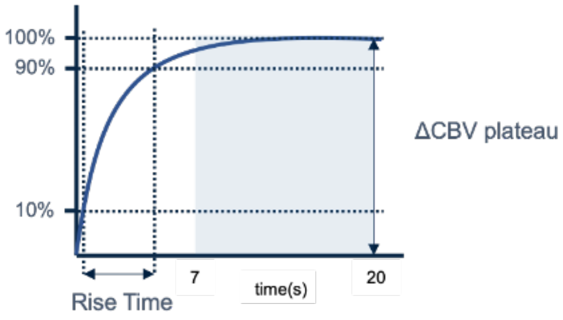

Supplementary figure 1: Schematics illustrating how the rise time was determined from evoked hemodynamic response induced by whisker stimulation.

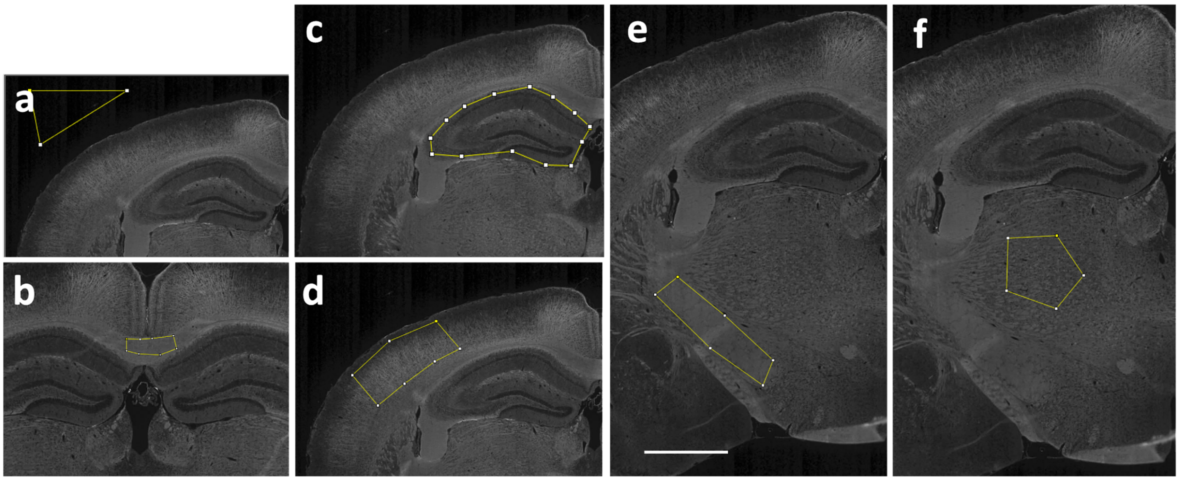

Supplementary figure 2: Brain areas contours for where MBP immunostaining quantification.

This figure displays the contours of the brain regions analyzed for MBP immunostaining quantification. Each panel highlights the specific area selected for measuring the mean gray value on each section: (a) Background, (b) Medial corpus callosum, (c) Hippocampus (d) S1BF (primary somatosensory cortex, barrel field), (e) Internal capsule, (f) Thalamus. These contours delineate the precise anatomical regions assessed to ensure consistent and accurate quantification across sections.

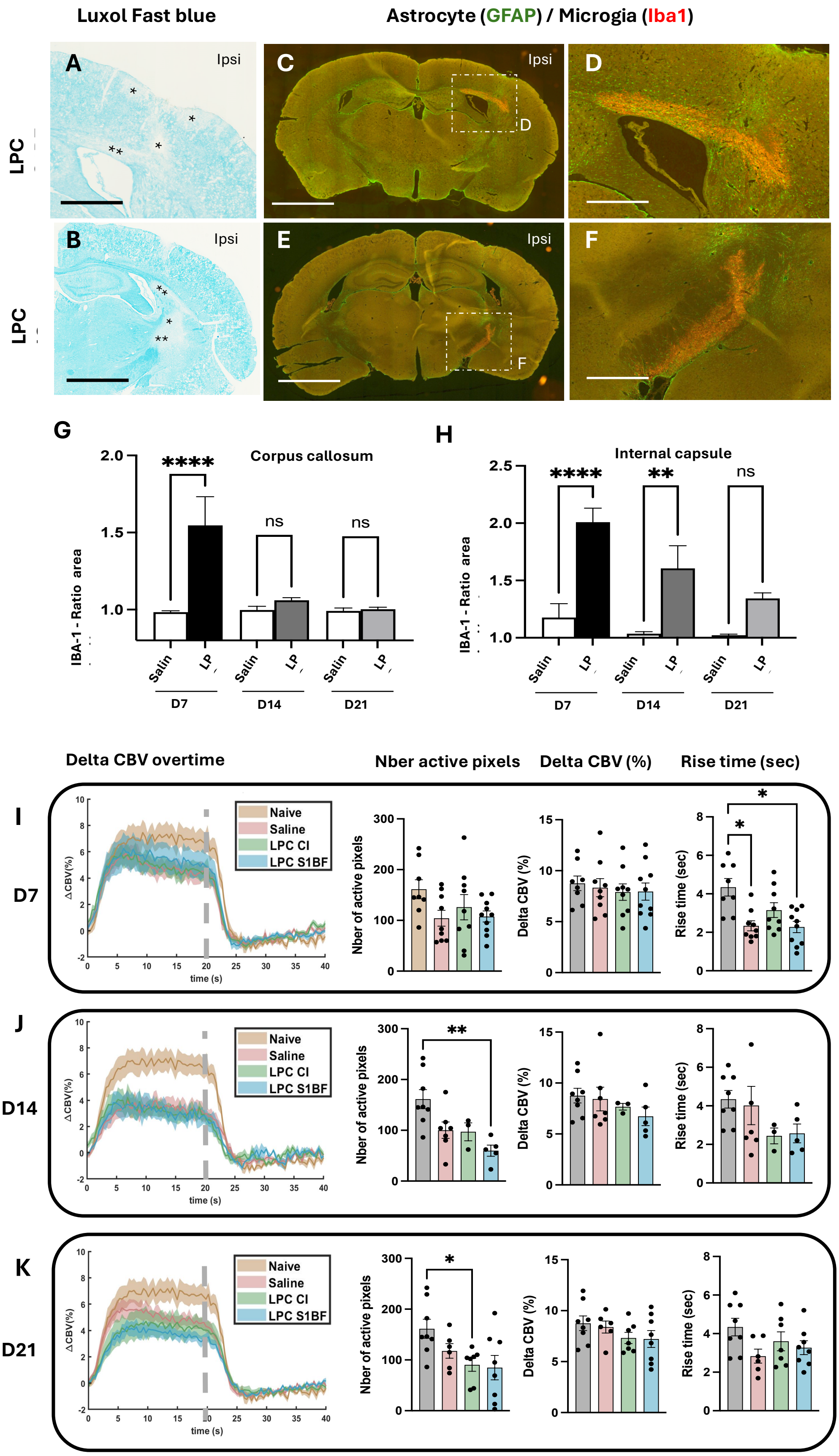
Luxol Fast blue
Astrocyte (GFAP) / Microgia (Iba1)
A
B
C
D
E
F
Ipsi
LPC
G
Corpus callosum
IBA-1 - Ratio area
****
ns
ns
Salin
LP
D7
D14
D21
H
Internal capsule
****
**
ns
Delta CBV overtime
Nber active pixels
Delta CBV (%)
Rise time (sec)
I
D7
J
D14
K
D21
Naive
Saline
LPC CI
LPC S1BF
ΔCBV(%)
time (s)
Nber of active pixels
Delta CBV (%)
Rise time (sec)
*
**

Supplementary figure 3: Localized unilateral demyelination induced by lysolecithin (LPC) does not alter the evoked hemodynamic response in the S1BF. This figure evaluates the impact of localized demyelination in the S1BF or internal capsule (IC) on the evoked hemodynamic response in the S1BF. LPC (1%, 1 µL) was injected unilaterally into the S1BF or IC, with controls receiving saline injections in the IC or no injections (naïve animals). Quantifications were performed at multiple time points (D7, D14, D21 post-injection; N=8 per group).

Luxol fast blue staining (A, B) confirmed the local demyelination in the cortex and corpus callosum (A, stars), 7 days after LPC post injection in the S1BF and in the dorsal portion of the internal capsule for the IC-injected animals (B). Consistent with previous observations in the literature, using a double staining Iba-1 (microglial cells) / GFAP (astrocytes, C-F), we confirm the presence of a strong microglial activation at the site of demyelination. Pictures in C-F show representative examples of this microglial recruitment in the corpus callosum (C, D) or IC (E, F) 7 days post-injection. D and F are higher power magnification of C and E, respectively. Scale bars: A: 0,8mm B: 1,5mm. C, E: 2mm. D, F: 1mm. G-H report the quantification of microglial recruitment in the corpus callosum (G) and internal capsule (H). Results are expressed as the mean ratio of area of Iba-1 staining (± SEM) in the ipsilateral versus the contralateral site. NS: non-significant, ** $p<0.01$, **** $p<0.0001$. A ratio higher than 1 is due to an increased microglial recruitment in the ipsilateral site compared to the contralateral.

I-K: Quantification of the functional hemodynamic response in the S1BF induced by whisker stimulation in groups of animals that received LPC injections either in the S1BF, the IC or saline injection in the IC. Similarly to figure 2, this figure presents from left to right i) the mean time-course of ΔCBV induced by whisker stimulation. ii) The number of active pixels, iii) The steady state CBV variation (ΔCBV, expressed in %, within the 7-20 sec stimulation interval) and finally the rise time. In all panels, the data are presented as mean ± SEM with an overlay of individual values. N=8 per group. * $p<0.05$, ** $p<0.01$.

Supplementary material #4: Materials and methods of the Supplementary figure 4:

Rational for the use of the LPC model

The lysolecithin (LPC) model consists in injecting the molecule directly in the spinal cord or the brain, resulting in a local, fast (a complete demyelination is observed in 7 days and reproducible demyelination and spontaneously remyelination in 4 weeks (El Behi et al., 2017; Luo et al., 2018)).

Based on the finding by Narayanan et al., that white or grey matter lesions (induced by LPC injections either in the internal capsule or the cortex, respectively) induce different functional auditory changes (Nouhoum et al., 2021), we used this approach to study the alterations of hemodynamic response in the S1BF evoked by whisker stimulations. In order to reduce the number of animals as much as possible (application of the '3R' European rule (reduce, replace, refine), only one injection control group was performed, consisting of stereotaxic injection of saline in the deepest region (the internal capsule). In order to evaluate the impact of the stereotaxic injection on the hemodynamic response, another control group was added for the whole study, in naïve animals.

The time course of demyelination was well established. To establish the impact of local demyelination at the peak of demyelination (D7) or at two time points during remyelination, we imaged different cohorts of animals, respectively at D7, D14 and D21.

Model development

Experiments were conducted using 2-3-month-old male C57BL/6 Rj mice (Janvier Labs, France). Anesthesia was induced by an intramuscular injection of a mixture of Ketamine (100 mg/kg) and Xylazine (20 mg/kg). If the animals reacted to a tail or paw pinch, an additional dose (10-15% of the initial dose) was given to deepen the anesthesia. To ensure animal welfare, 50 µL of Lidocaine (2mg/mL) was subcutaneously injected, and the skin was disinfected with Betadine before incision. The eyes were protected with a protective gel (Ocrygel). The head was mounted in a stereotaxic apparatus via ear bars. After unilateral craniotomy, lysolecithin (1%, 1 µL) was injected using a Hamilton syringe at the speed of 3 nL/s in the primary somatosensory cortex barrel field (S1BF; anteroposterior, −1.5 mm; lateral, 2.5 mm from bregma; dorsoventral, 1.5 mm from the brain surface) or in the internal capsule (IC; anteroposterior, −1.5 mm; lateral, 2.5 mm; dorsoventral, 3.5 mm). After suturing the skin, the animal was subcutaneously injected with 200 µL glucose

(25 mg/mL), 200 µL Metacam (0.5 mg/mL), and 100 µL Antisedan (2.1 mg/mL) for its well-being.

Reproducibility of the stereotaxic injections

To improve the reproducibility of the injection and remove user-dependent variability, we used the 'Brain positioning system' developed by Iconeus to perform our stereotaxic injections, with a prototype of syringe holder custom-made for this project. This technology uses the overlap of the 3D registration of the animal's vasculature in the template of the 3D volume of the Allen Brain Atlas (Nouhoum et al., 2021)).

After positioning of the animal on the stereotaxic frame, and stabilization (see above), as for the recognition of the plane of imaging, a linear 2D scan of the entire brain vasculature was conducted, and its alignment with the atlas was computed using the software Icoscan. Using the software Icostudio, the coordinates of injection (see above) were computed and the motors moved the syringe holder at these coordinates.

Double Iba-1-GFAP immunofluorescent staining and their quantification

Two slides (each one containing eight sections) of each animal were washed three times in 0.1M phosphate buffer with 0.9% NaCl (PBS). They were incubated overnight at room temperature with a mixture of primary antibodies: mouse anti-GFAP, (Merck-Sigma, Ref: MAB360, 1:1000), rabbit anti-Iba1, (Wako, Ref: W1W019-19741, 1:1000), diluted in 0.3% triton X-100. After the three washes, they were incubated for 2h in a mixture of secondary antibodies (Alexa Fluor 488-conjugated donkey anti-mouse antibody, 1:1000; Invitrogen and Alexa Fluor 594-conjugated donkey anti-rabbit antibody, 1:1000; Invitrogen). Finally, the sections were cover slipped using Fluoromount (Sigma, Aldrich). The sections from all animals were stained simultaneously.

Statistics

Data from the Iba-1 quantifications were analyzed using the Brown Forsythe and Welch ANOVA test, followed by Dunnett's T3 multiple comparison tests.

As performed in the rest of our study, the statistical analysis of the four descriptors of the hemodynamic response measured by fUS imaging (i.e. number of active pixels, rise time and variation of ΔCBV) across time was performed using a repeated measures one-way ANOVA, followed by Tukey's test for post-hoc multiple comparisons.

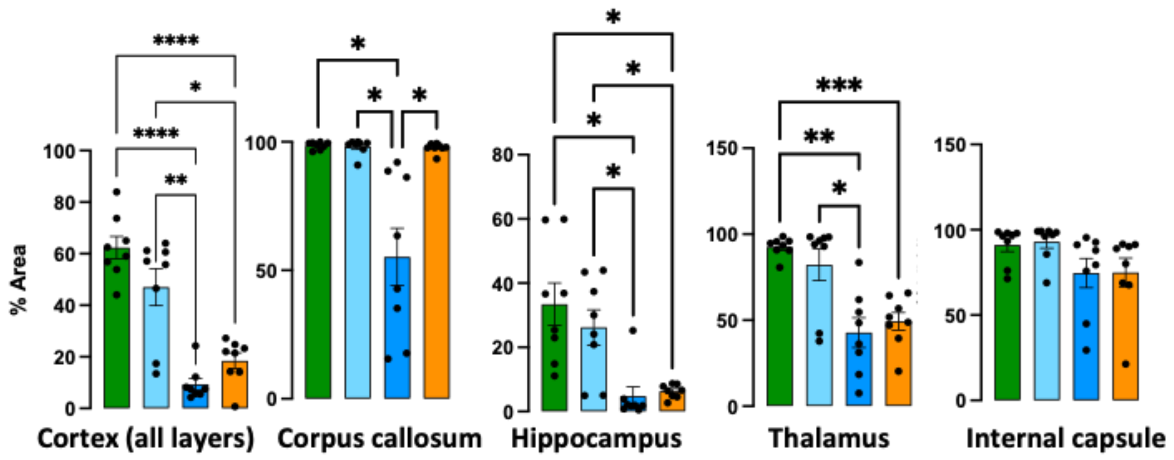

Supplementary figure 5: Quantification of MBP immunostaining changes using surface area analysis. This figure presents an alternative method of quantifying MBP immunostaining changes across the various experimental groups and time points studied. Instead of mean gray intensity, this analysis measures the percentage surface area occupied by MBP staining.

The surface area analysis yielded results consistent with the initial quantification based on mean grey intensity. This method corroborates the observed changes in myelin content across different brain regions and experimental conditions.

References

El Behi, M., Sanson, C., Bachelin, C., Guillot-Noël, L., Fransson, J., Stankoff, B., Maillart, E., Sarrazin, N., Guillemot, V., Abdi, H., Cournu-Rebeix, I., Fontaine, B., & Zujovic, V. (2017). Adaptive human immunity drives remyelination in a mouse model of demyelination. *Brain*, *140*(4), 967–980. doi: 10.1093/brain/awx008

Luo, Q., Ding, L., Zhang, N., Jiang, Z., Gao, C., Xue, L., Peng, B., & Wang, G. (2018). A stable and easily reproducible model of focal white matter demyelination. *Journal of Neuroscience Methods*, *307*, 230–239. doi: 10.1016/j.jneumeth.2018.05.024

Nouhoum, M., Ferrier, J., Osmanski, B.-F., Ialy-Radio, N., Pezet, S., Tanter, M., & Deffieux, T. (2021). A functional ultrasound brain GPS for automatic vascular-based neuronavigation. *Scientific Reports*, *11*(1), 15197. doi: 10.1038/s41598-021-94764-7
